# Supplementary material for: Overexpression of Mitochondrial IF1 Prevents Metastatic Disease of Colorectal Cancer by Enhancing Anoikis and Tumor Infiltration of NK Cells
Source: Cancers (Basel). 2019 Dec 19;12(1):22. doi: 10.3390/cancers12010022 (PMC7017164; doi:10.3390/cancers12010022)

*Supplementary material*

Overexpression of mitochondrial IF1 prevents metastatic disease of colorectal cancer by enhancing anoikis and tumor infiltration of NK cells

Lucia González-Llorente, Fulvio Santacatterina, Ana García-Aguilar, Cristina Nuevo-Tapioles, Sara González-García, Zuzana Tirpakova, María Luisa Toribio and José M. Cuezva*


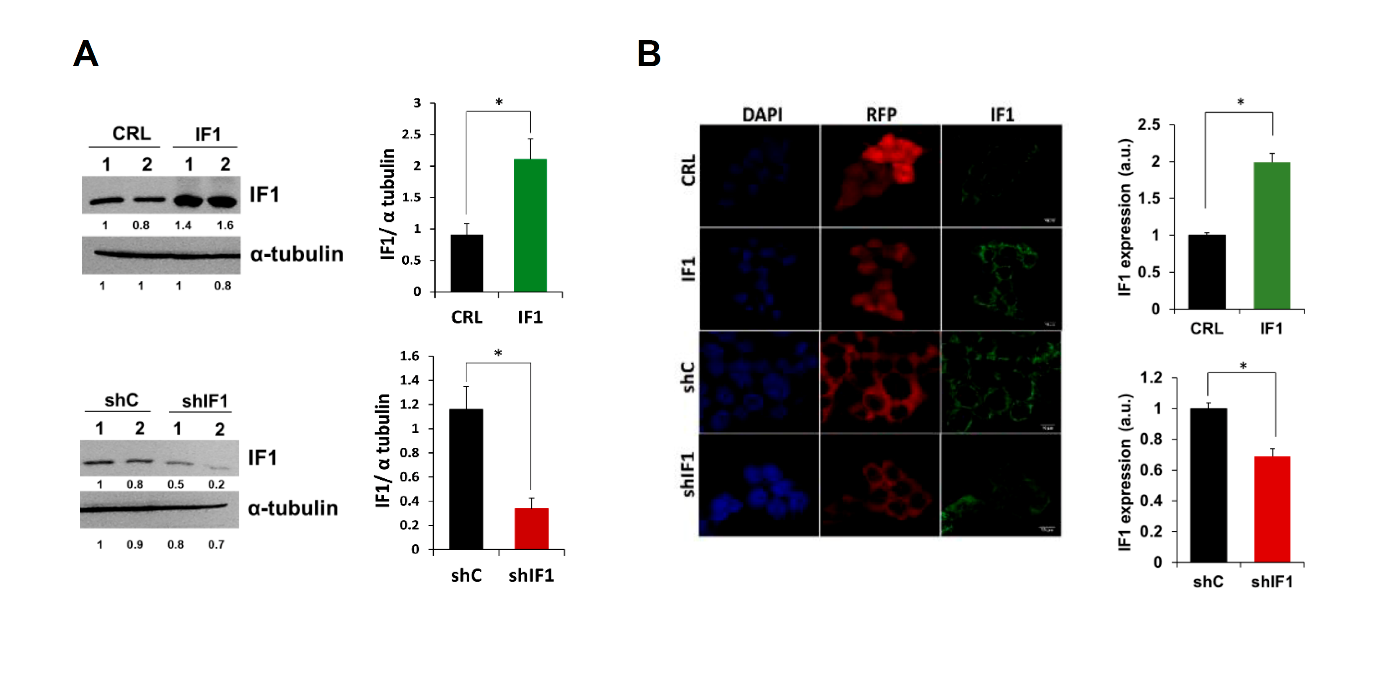


**Figure S1.** Development of stable HCT116 cell lines overexpressing (IF1) or silencing (shIF1) IF1. (**A, B**) The histograms show the quantification of IF1 expression by immunoblotting (**A**) and immunofluorescence microscopy (**B**). 1 and 2 are two different samples out of 5 different preparations. The results shown are mean ±S.E.M; *, p<0.05 when compared to its respective control. RFP, red fluorescent protein.


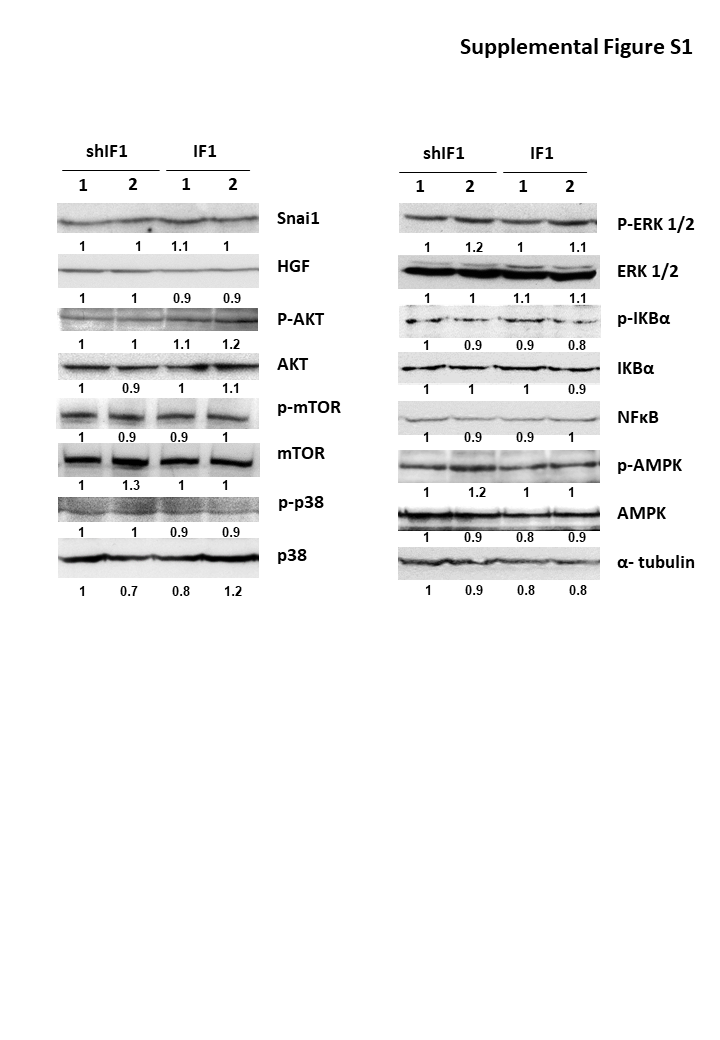


**Figure S2.** Western blots analysis of the expression of Snail and hepatocyte growth factor (HGF) proteins and the expression and phosphorylation of AKT, mTOR, p38, ERK1/2, IKBα, NFκB and AMPK proteins in two different replicates of IF1-silenced (shIF1) and IF1-overexpressing (IF1) HCT116 cells. α-tubulin is shown as loading control.


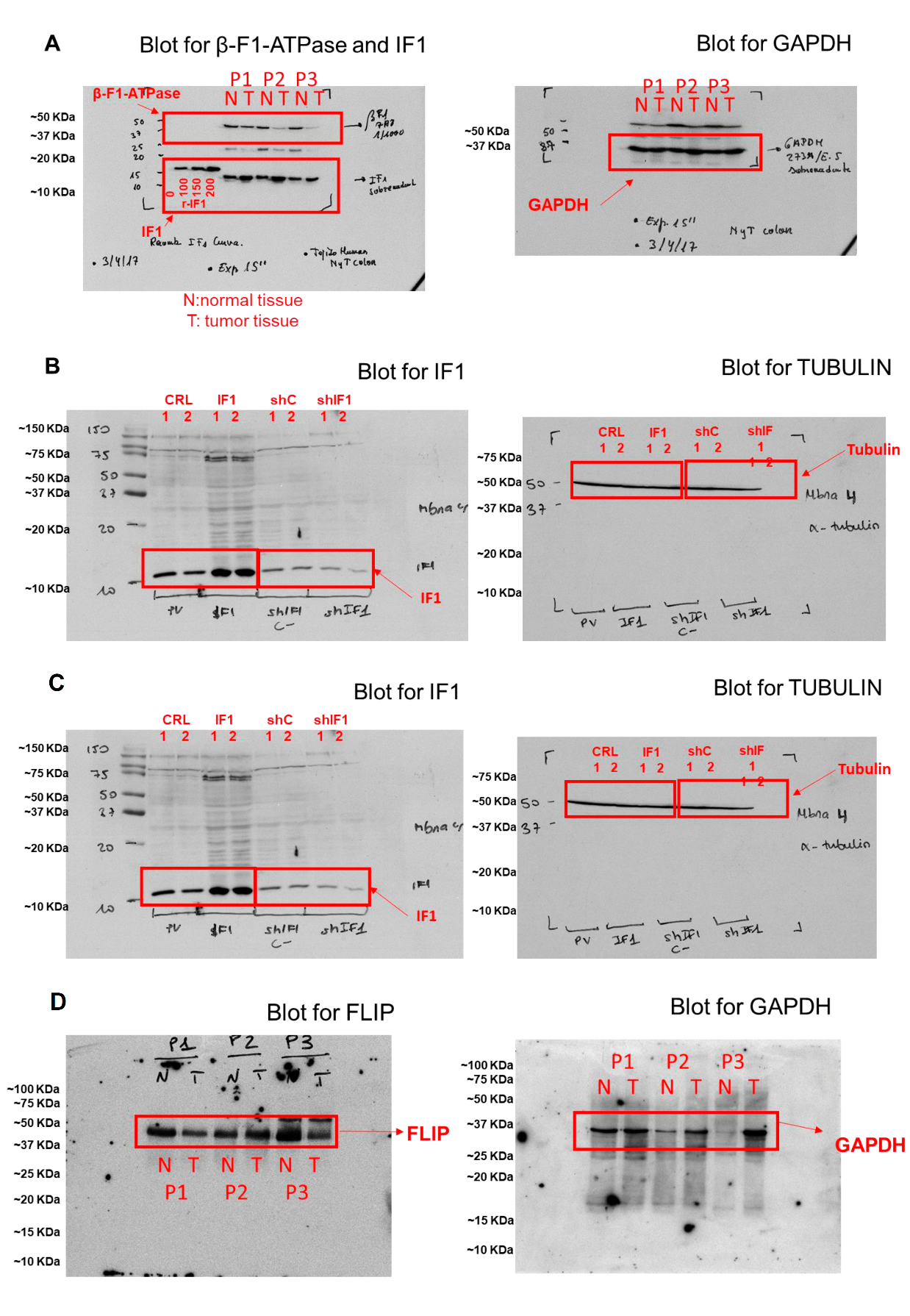


**Figure S3.** Full-length immunoblot images from Figures 1B, S1 and 6B, 6C. (**A**) Complete Western blot images of βF1-ATPase, IF1 and GAPDH in paired normal (N) and tumor (T) biopsies derived from three representative patients and increasing amounts of the recombinant IF1 (r-IF1) protein (0-200 ng) from Figure 1B. (**B**) Complete Western blot images of IF1 and tubulin as loading control. 1 and 2 are two different samples from Figure S1. (**C**) Complete Westerm blot images of FLIP and GAPDH as loading control from Figure 6B. 1 and 2 are two different samples. (**D**) Complete Westerm blot images of FLIP and GAPDH in paired normal (N) and tumor (T) biopsies derived from three representative patients from Figure 6C.


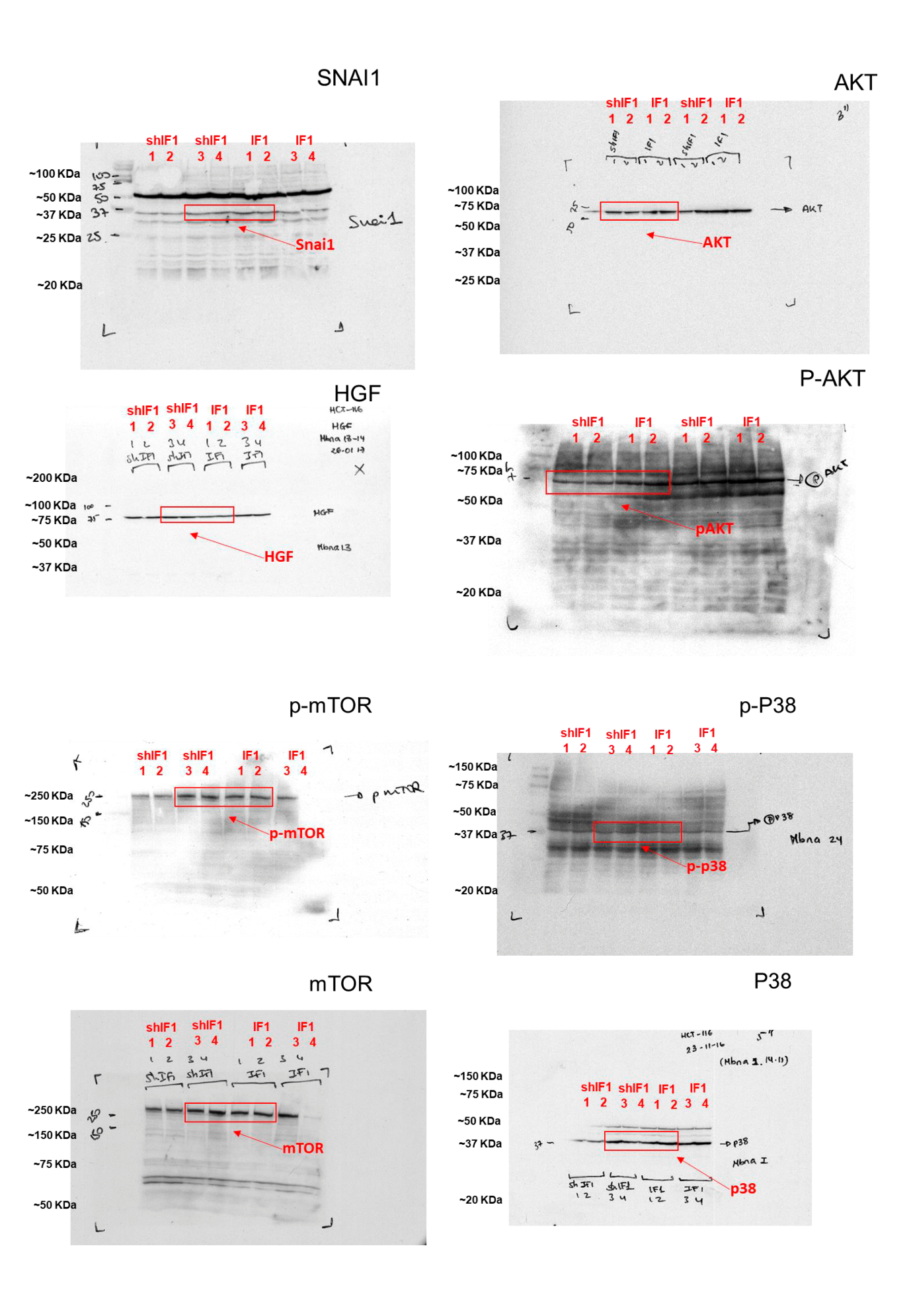


**Figure S4.** Full-length immunoblot images from left panel of Figure S2. Complete Western blot images of Snai1, HGF, p-mTOR, mTOR, AKT, p-AKT, p-p38 and p38. 1 and 2 are two different samples.


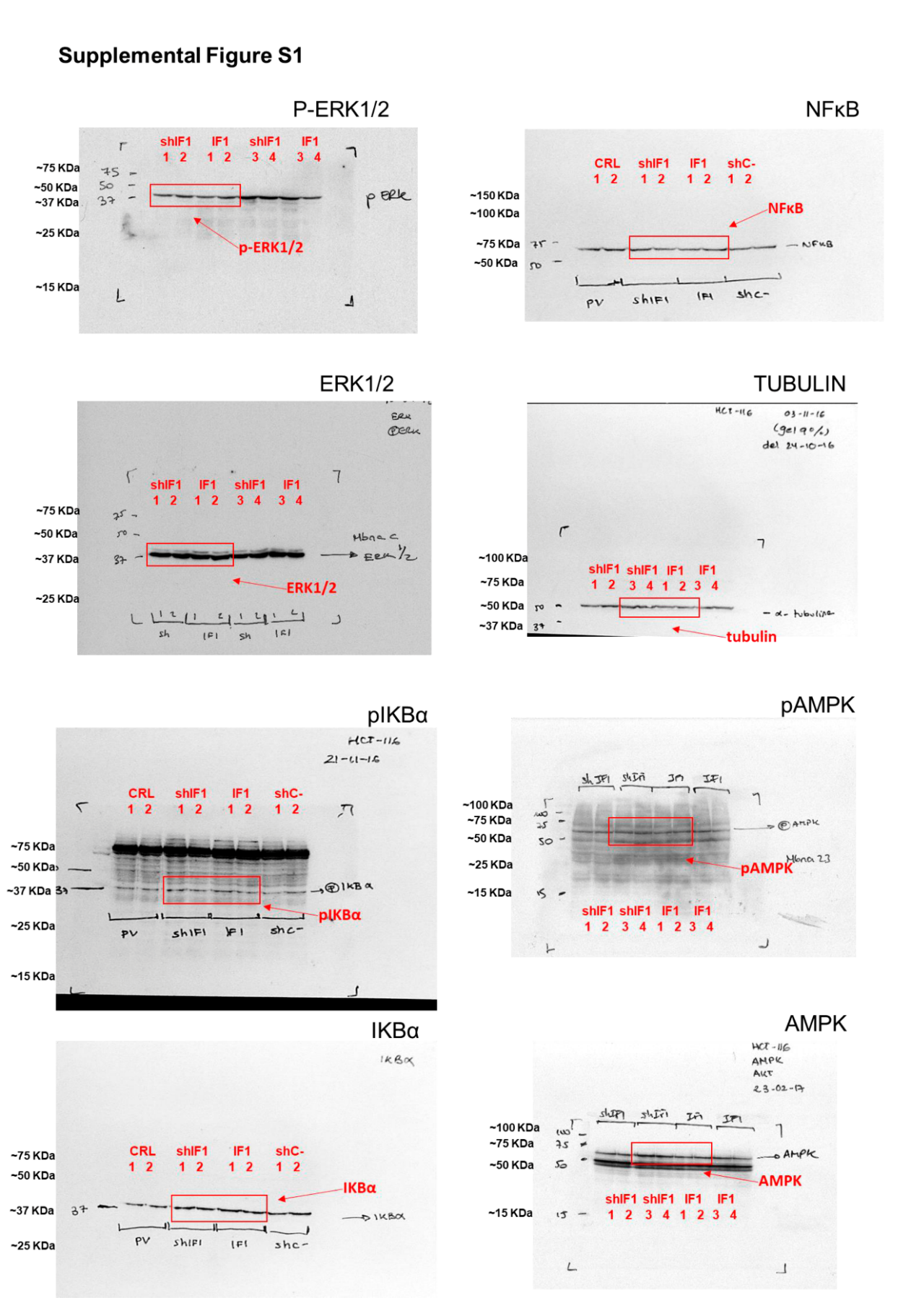


**Figure S5.** Full-length immunoblot images from right panel of Figure S2. Complete Western blot images of p-ERK1/2, ERK1/2, pIKBα, IKBα, NFκB, Tubulin, pAMPK and AMPK. 1 and 2 are two different samples.

© 2019 by the authors. Licensee MDPI, Basel, Switzerland. This article is an open access article distributed under the terms and conditions of the Creative Commons Attribution (CC BY) license (http://creativecommons.org/licenses/by/4.0/).
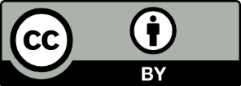

Supplement: Supplementary file 1 [file cancers-12-00022-s001.zip › Supplementary Information.docx]
